# Aortic rupture during transcatheter aortic valve replacement requiring emergent thoracic endovascular aortic repair and endograft infection requiring endograft explant

**DOI:** 10.1016/j.jvscit.2025.101746

**Published:** 2025-02-04

**Authors:** Ryan Wahidi, Dan Kindell, Puja Kachroo, J.Westley Ohman

**Affiliations:** a Department of Surgery, Section of Vascular Surgery, Washington University School of Medicine, St. Louis, MO; b Division of Cardiothoracic Surgery, Washington University School of Medicine, St. Louis, MO

**Keywords:** TEVAR, Infection, Explant, Aortobronchial, Fistula

## Abstract

Infection of endografts after thoracic endovascular aortic repair (TEVAR) is a catastrophic complication with dramatically high morbidity and mortality. We present the case of a 58-year-old gentleman who underwent TEVAR for aortic rupture during transcatheter aortic valve replacement, later presenting with TEVAR infection, endocarditis, and aortobronchial fistula who underwent TEVAR explantation, aortic valve replacement, and aortic reconstruction. The patient consented to publication of his operative course.

Thoracic endovascular aortic repair (TEVAR) has largely supplanted open repair as a first-line approach to pathologies of the descending thoracic aorta, used most frequently for thoracic aortic aneurysms and type B aortic dissections.^1^^,^^2^ Blunt traumatic injury and transections of the thoracic aorta may also be addressed by TEVAR.^3^, ^4^, ^5^ Infection of TEVARs often present months to years after the index procedure with nonspecific symptoms, including pain, fevers, and chills.^6^ This report details the use of TEVAR for iatrogenic thoracic aortic trauma during transcatheter aortic valve replacement (TAVR), and successful surgical management of endograft infection 7 months after the index operation.

## Case report

**Fig 1:**
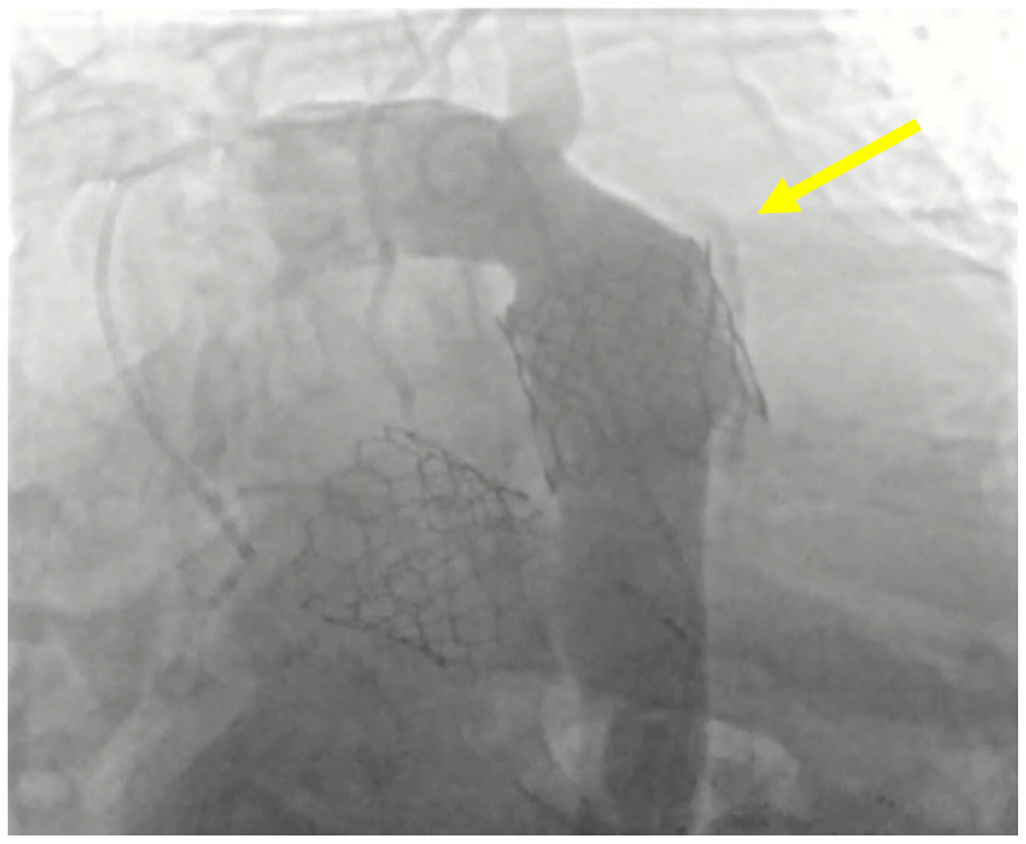
Intraoperative angiogram at index procedure before thoracic endovascular aortic repair (TEVAR) placement. Maldeployed Sapien 3 ultra valve was pulled back into descending thoracic aorta, and additional valve was adequately placed. Periaortic contrast (*yellow arrow*) is seen around the distal valve with concern for contained aortic rupture in the setting of intraoperative hemodynamic instability.

The patient is 58-year-old man with a history of coronary artery disease who previously underwent percutaneous coronary intervention; he has congestive heart failure, end-stage renal disease on peritoneal dialysis, and severe aortic stenosis; he was scheduled to undergo TAVR as opposed to open repair to minimize risk of postoperative hemodialysis requirement. The TAVR (29 mm Sapien 3 ultra) procedure was complicated by valve dislodgement and thoracic aortic rupture immediately distal to the left subclavian artery. The patient underwent emergent TEVAR (overlapping 34 × 100 mm and 31 × 100 mm GORE cTAGs; W. L. Gore & Associates, Flagstaff, AZ) extending from zone 2 to zone 5 with left subclavian coverage and was admitted to the intensive care unit (ICU). Angiography demonstrating the location of rupture is presented in Fig 1. Postoperatively, he developed left upper extremity ischemia for which he underwent laser fenestration and stenting of his left subclavian artery from an open brachial approach on the first postoperative day. An emergent subxiphoid pericardial window was required on the second postoperative day to relieve cardiac tamponade. He was later extubated, transitioned back to peritoneal dialysis, and discharged in stable condition. In the interim, he underwent pacemaker placement at an outside hospital owing to the development of second-degree AV block.

**Fig 2:**
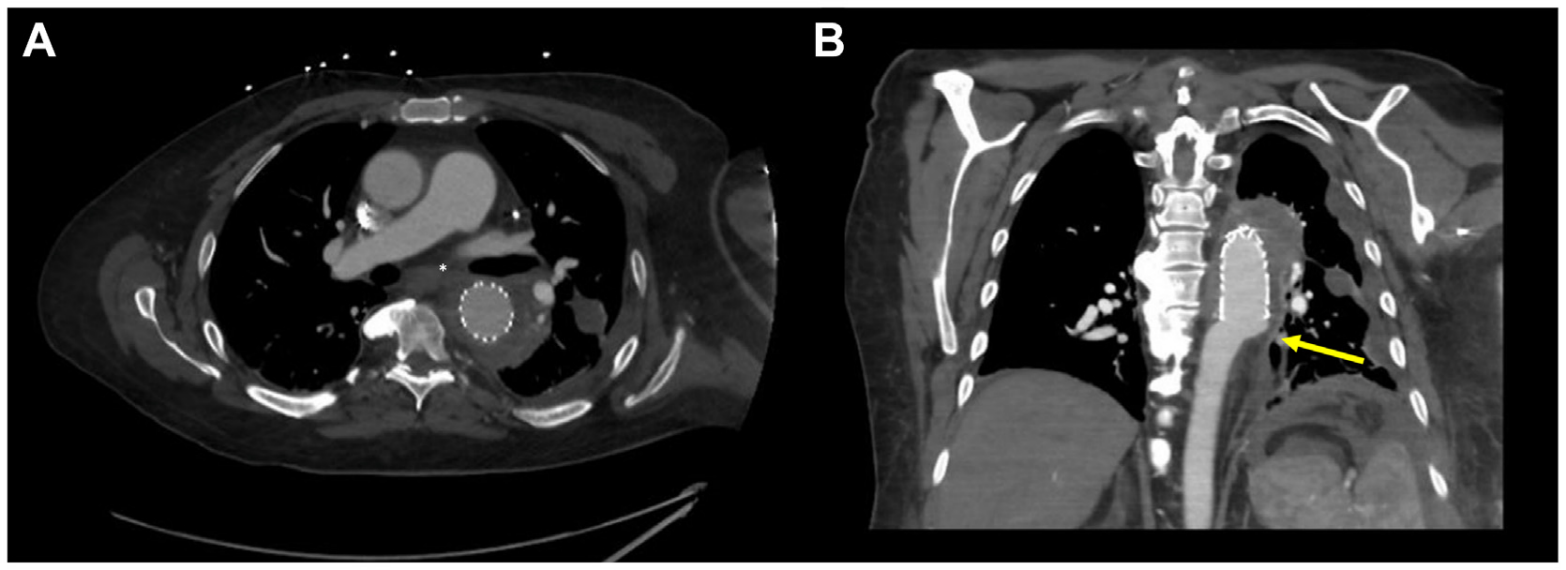
Patient presentation to emergency department following development of hemoptysis. **(A)** Axial cross-section demonstrating severe aortic wall thickening in the proximity of the left mainstem bronchus (asterisk). **(B)** Coronal view with concern for poor distal seal and possible endograft migration (*yellow arrow*).

Seven months after his index operation, he presented to the emergency department with hemoptysis. Computed tomography imaging demonstrated TEVAR migration with periaortic hematoma vs pseudoaneurysm, with concern for an aortobronchial fistula (ABF) (Fig 2). He was emergently taken to the hybrid suite and the lesion was covered with a tapered 31 × 26 mm GORE TAG. The previous TEVAR was relined with a 34 mm GORE TAG; notably, his prior left subclavian stent had been thrombosed chronically without evidence of left upper extremity ischemia and was, therefore, covered. Given the wall thickening and concern for ABF, vancomycin and cefepime were started empirically.

Bronchoscopy revealed persistent pooling of blood in the superior segment of the left lower lobe, concerning again for ABF. Blood cultures were positive for methicillin-resistant *Staphylococcus epidermidis* and a transesophageal echocardiogram was performed to evaluate the AVR. A transesophageal echocardiogram was concerning for a 1.7-cm vegetation on the AVR. A staged operative intervention was planned to resect and replace the infected valve and bypass the ascending and thoracic aorta, followed by revascularization of the left upper extremity, and finally excision of the infected TEVARs.

The staged operative intervention occurred over 3 days. First, the patient was taken for a sternotomy, the pacemaker was explanted, and a grossly infected TAVR was encountered with associated aortic root abscess. The patient was heparinized and placed on cardiopulmonary bypass for approximately three hours; neuromonitoring was performed with cerebral oximetry. The aortic valve was explanted, a 25-mm Edwards Inspirus valve was sewn into place, and the aortic root was reconstructed with bovine patch. A 16-mm rifampin-soaked Dacron graft was sewn end-to-side to the proximal ascending aorta, and from this, a 6-mm graft was sewn end-to-end to the left common carotid and a 12-mm graft was sewn end-to-end to the innominate. The left common carotid and innominate were then ligated proximally. The supraceliac aorta was exposed in the standard fashion after extending the sternotomy. A cruciate incision was made in the diaphragm just medial to the inferior vena cava and the graft was tunneled and sutured end-to-side to the supraceliac aorta. The ascending aorta was then ligated and oversewn with felt and bovine pericardium. For the second stage, a left carotid-subclavian transposition was performed in preparation for distal thoracic aorta ligation. For the third stage, a left posterolateral thoracotomy was performed, and the 5th and 8th intercostal spaces entered. The descending aorta was ligated proximal to the supraceliac graft anastomosis and covered with a bovine pericardium patch. The descending aorta was entered and the TEVARs explanted and sent for culture along with aortic tissue. The thoracotomy was closed in standard fashion and the patient was returned to the ICU.

**Fig 3:**
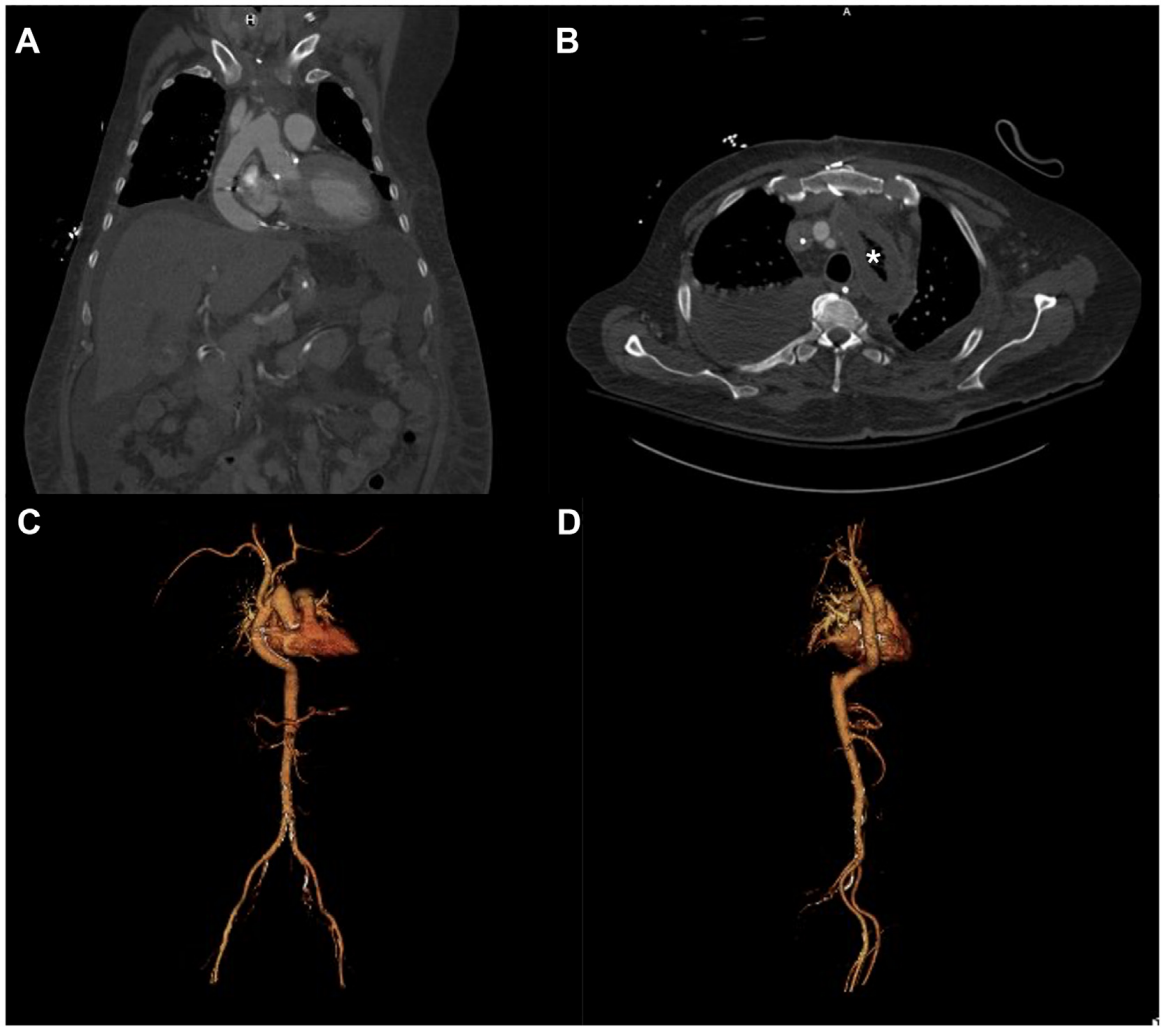
Postoperative imaging obtained 3 weeks after staged endograft and transcatheter aortic valve replacement (TAVR) explantation with extra-anatomic reconstruction. **(A)** Course of the patent bypass graft from the ascending aortic graft tunneled through the diaphragm to the supraceliac aorta. **(B)** A portion of excluded remnant aorta can be seen (marked with asterisk) with associated wall thickening is shown. **(C** and **D)** A three-dimensional reconstruction of the patient's final anatomy is presented.

Cultures from TEVARs and aortic tissue were positive for methicillin-resistant *S epidermidis*, and the patient was continued on a course of vancomycin for 6 weeks with lifelong oral suppression. The patient was extubated on postoperative day 9; however, reintubation was required that same day, and 2 weeks postoperatively the patient underwent tracheostomy placement. Continuous renal replacement therapy was transitioned to intermittent hemodialysis, and the patient was transferred out of the ICU on postoperative day 31, and ultimately discharged to an extended care facility after 71 days. Computed tomography angiography images obtained 3 weeks after the staged operation are shown in Fig 3, demonstrating stable postoperative changes and patent bypass graft. After discharge, the patient elected to pursue follow-up at another institution closer to their home; computed tomography imaging obtained 1 year after the surgical repair remains stable.

## Discussion

Endograft infection is a rare and challenging delayed complication of TEVAR, with a reported occurrence ranging between 0.6% and 4.0%.^7^^,^^8^ Owing to its rarity, the available literature remains relatively sparse and limited to case reports and series, leading to a lack of well-established management guidelines. Infected TEVARs often clinically manifest as nonspecific, persistent constitutional symptoms, or as in this case, may present after fistualization—aortobronchial or aortoesophageal—has occurred with associated hemoptysis or hematemesis.^9^ Given the increased incidence of endograft usage and an aging population, the incidence of endograft infection may follow suit, becoming increasingly relevant to a contemporary vascular practice.^10^

The present case stands out owing to the unusual initial iatrogenic aortic injury necessitating TEVAR as a precipitating factor, further complicating the final explant owing to the need for concurrent aortic valve explant and repair. More important, it underscores the need for comprehensive interdisciplinary care in the management of patients with thoracic endograft infections; in the case of this patient, the role of several combined surgical cases with vascular and cardiac surgery.

Treatment options for infected TEVARs are broadly endograft preserving with suppressive antibiotics or endograft explantation; while both are associated with high inpatient mortality, 43% and 37%, respectively; explantation remains the definitive treatment option, particularly for younger, healthier patients as described the present case.^9^ The presence of a fistula necessitates repair and portends higher perioperative mortality, 51.5% on recent meta-analysis.^8^^,^^9^ Aortic reconstruction options following TEVAR explantation are principally in line or extra-anatomic; a meta-analysis of these surgical approaches found no significant perioperative mortality difference between approaches.^9^ An extra-anatomic approach is preferred in our practice in the setting of any aerodigestive-aortic fistula, as was in the case in this patient. Expected functional outcomes for patients following TEVAR explantation remains poorly described.

## Conclusions

As the incidence of TEVAR continues to increase, the incidence of infected TEVARs may follow suit. The present case describes the use of TEVAR emergently for an iatrogenic aortic rupture, and staged extra-anatomic reconstruction with endograft explantation as a durable surgical option for endograft infection.

## Funding

None.

## Disclosures

J.W.O. is a consultant for W. L. Gore & Associates, Cook, TerumoAortic, and Globus.
